# P391: Audit of the processing of medical devices in reusable surgical services of the university hospital Yalgado Ouedraogo (Chu-Yo)

**DOI:** 10.1186/2047-2994-2-S1-P391

**Published:** 2013-06-20

**Authors:** J Josephine, B Ndoye, IP Guissou, FS Zongo, H Dehainsala

**Affiliations:** 1 RIPAQS Burkina, Ouagadougou, Burkina Faso; 2 PRONALIN, Dakar, Senegal

## Introduction

In surgical services, reusable equipment must follow a strict process with several stages (pre-disinfecting, cleaning, rinsing, drying, packaging and sterilization).

Following the steps of this process is essential for the prevention of infections associated with surgery and poses a problem at CHU-YO since it does not have central sterilization department as recommended.

## Objectives

Conduct an audit of sterilization practices in surgical services at CHU-YO to propose a plan for overall improvement.

## Methods

Descriptive cross-sectional survey conducted in seven surgical services of four months from 04 December 2009 to 2 April 2010, without any prior interventions; Collection of data from personnel involved in the treatment of surgical equipment for the sterilization using a self-administered questionnaire and an observation of actions of disinfection.

## Results

i) Inadequate organization of work, ii) Equipment obsolete iii) Absence of pocket cards, protocols and training of frontline personnel) Pre-disinfection: 93.3% immediately after the intervention, v ) Cleaning: 4.65% of cases, change the cleaning solution in 60.5% of cases, vi) Rinse: no rinsing between pre-cleaning and disinfecting, rinsing after cleaning only.

## Comments

High rates of non-compliance practices in surgical services; Need for a plan of action to organize the work of processing equipment for the appointment of staff to different responsibilities and different steps to establish appropriate protocols at all stages, to train frontline staff these protocols to implement protocols and evaluate regularly;

This action plan should be accompanied by monitoring infection rates, to judge its effectiveness.

## Conclusion

Magnitude of the task in unstructured health; Need to develop human resources in hospital hygiene and set up committees to fight against HAI.

## Disclosure of interest

None declared

